# GTP-Bound N-Ras Conformational States and Substates Are Modulated by Membrane and Point Mutation

**DOI:** 10.3390/ijms25031430

**Published:** 2024-01-24

**Authors:** Alexandra Farcas, Lorant Janosi

**Affiliations:** Department of Molecular and Biomolecular Physics, National Institute for Research and Development of Isotopic and Molecular Technologies, 67-103 Donat Street, 400293 Cluj-Napoca, Romania; alexandra.farcas@itim-cj.ro

**Keywords:** molecular dynamics, Ras protein, N-Ras, Ras states, plasma membrane, lipid bilayer, conformational states, point mutation, G12V

## Abstract

Oncogenic Ras proteins are known to present multiple conformational states, as reported by the great variety of crystallographic structures. The GTP-bound states are grouped into two main states: the “inactive” state 1 and the “active” state 2. Recent reports on H-Ras have shown that state 2 exhibits two substates, directly related to the orientation of Tyr32: toward the GTP-bound pocket and outwards. In this paper, we show that N-Ras exhibits another substate of state 2, related to a third orientation of Tyr32, toward Ala18 and parallel to the GTP-bound pocket. We also show that this substate is highly sampled in the G12V mutation of N-Ras and barely present in its wild-type form, and that the G12V mutation prohibits the sampling of the GTPase-activating protein (GAP) binding substate, rendering this mutation oncogenic. Furthermore, using molecular dynamics simulations, we explore the importance of the membrane on N-Ras’ conformational state dynamics and its strong influence on Ras protein stability. Moreover, the membrane has a significant influence on the conformational (sub)states sampling of Ras. This, in turn, is of crucial importance in the activation/deactivation cycle of Ras, due to the binding of guanine nucleotide exchange factor proteins (GEFs)/GTPase-activating proteins (GAPs).

## 1. Introduction

Ras proteins are well-studied small GTPases that function as molecular switches between GTP-bound active and GDP-bound “inactive” forms to mediate signal transduction pathways that regulate cell growth, differentiation, and proliferation [1,2]. Now it is known that Ras proteins are post-translationally modified membrane-bound proteins that form non-overlapping, dynamic, nano-sized domains (nanoclusters) in an activation state-/isoform-dependent manner [3,4]. These nanoclusters act as binary switches [3,5,6], whose activation is aided by guanosine exchange factors (GEFs), which stimulate the dissociation of GDP and subsequent binding of GTP (GTP is ∼9-fold more abundant in the cytosol than GDP [7]). GTP-bound Ras activates a great variety of downstream signaling pathways by interacting with many effectors [8,9]. Ras signaling is terminated by the hydrolysis of the bound GTP, which can be accelerated by several orders of magnitude by GTPase-activating proteins (GAPs) [10]. Oncogenic point mutations are resistant to GAP binding and, hence, render them constitutively active. Most importantly, these mutations are associated with ∼30% of all cancers, and in some specific cancers, they are found in over 90% of cases [11,12], as well as developmental disorders [13,14]. Nonetheless, little success has been achieved thus far in developing clinically effective targeted therapy using oncogenic point mutation sites [15].

H-, N-, and K-Ras are three isomers that are ubiquitously expressed in humans. While they share nearly identical catalytic machinery (G-domain variations are less than 15%), their C-terminal hypervariable region (HVR), which contains the lipid modifications (lipid anchor), is ∼85% different. The mechanism by which the membrane-binding motif attaches to cellular or model membranes was extensively investigated both experimentally (reviews in Refs. [16,17]) and computationally [18,19,20,21,22,23,24,25]. Nonetheless, this knowledge alone is not enough to explain the complexity of the spatial and temporal organization of the three isomers into non-overlapping nanoclusters on the plasma membrane surface [3,6,26]. Further experiments on full-length proteins [27,28,29,30,31], as well as on simplified model peptides [32,33,34,35], have led to the consensus that H-Ras prefers liquid-ordered domains, N-Ras prefers the border between the liquid-ordered ( $L_{o}$ ) and liquid-disordered ( $L_{d}$ ) domains, while K-Ras prefers the $L_{d}$ domain. Computer simulations have shed light on the role and importance of lipid anchors [36] in the localization of the isomers, and have highlighted the effects of peptide concentration and lipid composition on the formation and domain partitioning of the anchor peptides into nanoclusters [37].

Extensive experimental structural studies on very similar isomers have led to an impressive database of Ras structures (predominantly H- and K-Ras). However, all these crystallographic structures show that Ras proteins exhibit multiple conformational states, mainly driven by the so-called switch I and switch II regions. Active (GTP-bound) conformations can be grouped into two main states at dynamic equilibrium: the “inactive” state 1 and “active” state 2 [38]. State 1 interacts with GEFs and, therefore, is characterized by an increased protein surface area (due to the opening in the nucleotide-binding pocket, required for the interaction with GEFs) [39], which, in turn, leads to higher switch I flexibility [40]. At the molecular level, the major characteristic of state 1 was shown to be the broken hydrogen bond between Thr35 and the ${\mathrm{Mg}}^{2+}$ ion [41]. State 2, on the other hand, can interact with a variety of effectors [39,42]. Recently, Li et al. [43] proposed that, at least for H-Ras, state 2 is in fact split into two separate conformational substates, corresponding to the Ras–effector interaction, and Ras–GAP interaction, respectively. The structural discrepancies between the two substates are minor. The main distinction is characterized only by the different orientations of the Tyr32 residue: toward the GTP pocket (Tyr32_*in*_) and toward the bulk solution (Tyr32_*out*_). The latter orientation is in agreement with Tyr32’s position in the crystallographic structure of Ras–GAP structures, required for the GAP Arg finger insertion needed for enhanced hydrolysis.

In fact, not only are the sampled GDP/GTP-bound states different, but their substates are also membrane-modulated and dependent on catalytic domain orientation [22,40,42,44,45]. The existence of additional substates has been further investigated in recent years. Hence, Chen et al. revealed that the K-Ras-GDP· ${\mathrm{Mg}}^{2+}$ product state has multiple stable substates in solution, suggesting that complexation with GEFs may involve a conformation-selection mechanism [46]. The use of pressure-induced crystallo-phase transitions provided a unique opportunity to investigate the structural determinants involved in the switching between Ras allosteric substates, without the need for mutations or external partners [47]. Since distinguishing between “active” and “inactive” molecular conformations is still very challenging, novel computational approaches have addressed this by using novel classification methods [48], free energy approaches [46], or density-based machine learning algorithms to cluster switch I and switch II loops into novel conformational subsets [49].

While a great amount of work has focused on H- and K-Ras, too little has been directed toward the study of N-Ras structural and conformational characteristics, although oncogenic N-Ras is the major cause of malignant melanomas, thyroid carcinomas, and some types of leukemia [12,50], with Gly12 being one of its most important oncogenic mutations [12]. Hence, an order of magnitude fewer crystallographic structures are available for N-Ras (e.g., GDP-bound N-Ras (3CON) [51] and GTP-equivalent GppNHp-bound N-Ras (5UHV) [52]). While it has been shown that the Ras anchor plays a major role in localization and, hence, differentiation of the Ras isomers [36,37], most of the work on structural Ras has focused on solvated Ras. Nonetheless, previous work has identified two membrane-bound catalytic domain Ras orientations (one parallel and one at an angle) [22], both nucleotide state- and isomeric-dependent [22,44,45]. Moreover, the G12V mutation was shown to adopt the parallel orientation for H-Ras [22]. However, it is still not clear how these findings can be applied to N-Ras.

Hence, in this paper, we shift our focus to N-Ras and, by means of molecular dynamics simulations, we show that *GTP-bound N-Ras* (i) exhibits three, Tyr32 orientation-characterized, conformational substates of state 2—the novel substate, has a quasi-parallel orientation of Tyr32 to the GTP-bound pocket, toward Ala18 ( ${\mathrm{Tyr}32}_{parallel}$ ); (ii) membrane anchoring to a plasma membrane model provides increased stability of the N-Ras system and, therefore, the accuracy of the distribution of conformational states and substates (state 1 and three substates of state 2) of N-Ras is strongly enhanced by the membrane presence; (iii) the ${\mathrm{Tyr}32}_{parallel}$ substate is barely sampled by the membrane-bound wild-type N-Ras; (iv) G12V mutation inhibits the GAP-bonded corresponding substate of state 2 ( ${\mathrm{Tyr}32}_{out}$ ), which is required for enhanced hydrolysis of GTP, rendering it oncogenic.

## 2. Results

**Figure 1:**
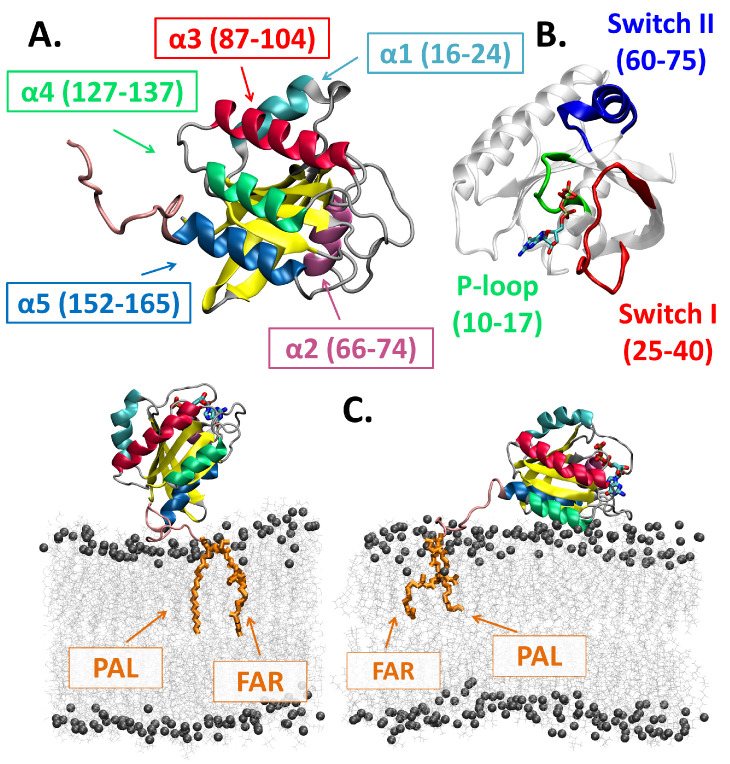
**N-Ras protein.** (**A**) The tertiary structure of the N-Ras protein. The five α -helices are colored in different colors, β -sheets are in yellow, and loops are in gray. (**B**) The functionally important regions, switch I (red) and switch II (blue), the phosphate-binding P-loop (green), and the guanosine triphosphate (GTP) molecule (atom-type-colored licorice representation). (**C**) The two initial configurations used for N-Ras were at ∼45° with the membrane (bottom left) and quasi-parallel to the membrane (bottom right). (Structure color-coding, as in (**A**), GTP representation and color-coding as in (**B**).) Cysteine palmitoyl and farnesyl lipid modifications (PAL and FAR, respectively, shown in orange licorice representation) anchor N-Ras to the membrane model (in gray, with P atoms in bead representation).

**Figure 2:**
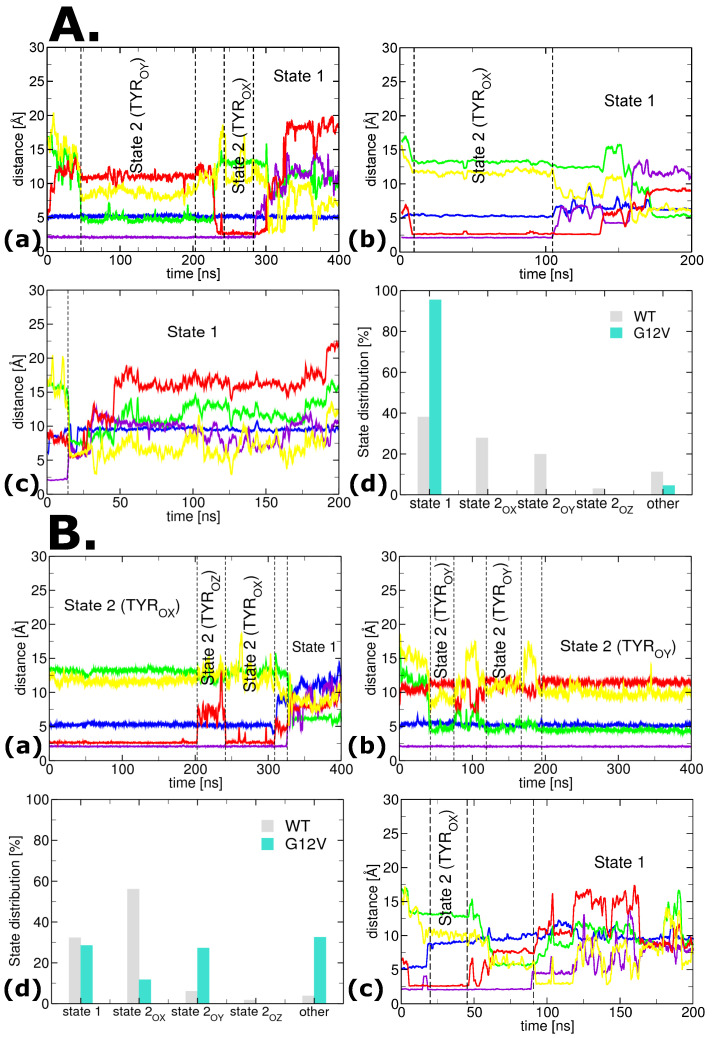
**Time evolution of representative simulation trajectories and the conformational distribution of (sub)states of N-Ras, as calculated over all the performed simulations.** (**A**) **In solution, without membrane**: time evolution for WT (a,b) and G12V (c), and distribution of (sub)states (d); (**B**) **Inserted in/bound to model membrane**: time evolution for WT (a) and G12V (b,c), and distribution of (sub)states (d). Time evolution plots represent the system dynamics, characterized by specific distances, involving the following atoms: amino acid backbone oxygen (O), γ oxygen of GTP ( $O_{\gamma }$ ), terminal oxygen at the end of Tyr ring (OH), sidechain oxygen of Thr (OG), magnesium (Mg). Subscript numbers indicate the residue to which the atoms belong. The distances are color-coded as follows: (i) $O_{12}$ - $O_{\gamma }$ in blue, (ii) ${\mathrm{OH}}_{32}$ - $O_{18}$ in green, (iii) ${\mathrm{OG}}_{35}$ -Mg in violet, (iv) ${\mathrm{OH}}_{32}$ - $O_{\gamma }$ in red, and (v) ${\mathrm{OH}}_{32}$ - ${\mathrm{OH}}_{40}$ in yellow.

The first set of MD simulations was performed for N-Ras (see Figure 1) wild-type (WT) in water with salt at physiological concentration, without membrane (see Figure 2A). Since most experimental and modeling studies on conformational state definitions [38,41,43] were conducted in similar conditions, we used these simulations as a reference for characterizing state 1 and state 2 (and their corresponding substates) at the molecular level.

**State 1 “inactive” state.** We used the relatively loose characterization of Muraoka et al. for the “inactive” state 1 at the molecular level, i.e., the conformation where the Thr35 hydrogen bond to ${\mathrm{Mg}}^{2+}$ ion is broken [41] (i.e., the violet line values in the trajectories increase dramatically Figure 2).

**Figure 3:**
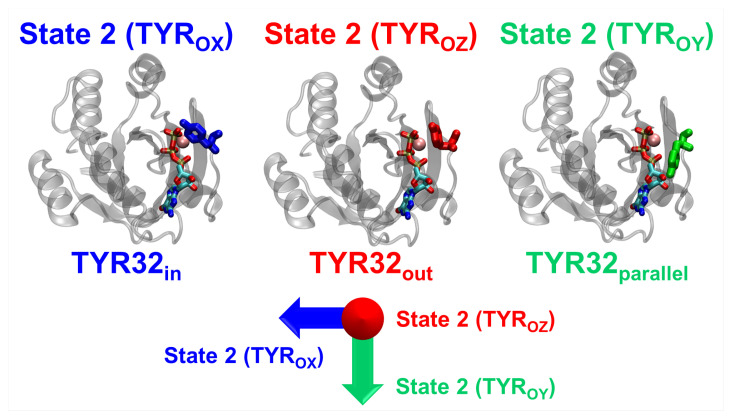
**N-Ras exhibits three substates of state 2.** These are mainly characterized by the quasi-perpendicular orientations of Tyr32. Protein is shown in the secondary structure representation in gray, GTP in the atom-type coded colors.

**State 2 exhibits three substates.** This is the “active” state, in which Thr35 binding to ${\mathrm{Mg}}^{2+}$ and GTP is crucial [38]. State 2 was shown to exhibit two substates for H-Ras, corresponding to two different functions [43]. Their major conformational difference involves the different orientations of Tyr32: OX and OZ [43]. In our simulations, we found that N-Ras exhibits, besides the above-mentioned orientations, a third *novel* orientation of Tyr32, OY , toward Ala18. All three have quasi-perpendicular orientations, as shown in Figure 3. Substate $2_{OX}$ features the Tyr32 sidechain oriented toward Gly12 and, therefore, toward the GTP-binding pocket; substate $2_{OY}$ has the Tyr32 sidechain oriented parallel to this pocket, toward Ala18, while substate $2_{OZ}$ has the Tyr32 sidechain oriented toward the bulk water (perpendicular to the plane formed by the other two directions). In the latter state, Tyr32 corresponds to its position when GAP is bound to Ras (PDB id 1WQ1 [53]).

### 2.1. Characterization of the Conformational (Sub)States

To monitor how substates shift, one approach is to investigate the conformational changes in the residues surrounding the specific region of interest during the simulation. This helps in understanding how the dynamics of these residues may influence the overall behavior of the system. Figure 2 depicts the distances between (i) $O_{12}$ - $O_{\gamma }$ in blue, (ii) ${\mathrm{OH}}_{32}$ - $O_{18}$ in green, (iii) ${\mathrm{OG}}_{35}$ -Mg in violet, (iv) ${\mathrm{OH}}_{32}$ - $O_{\gamma }$ in red, and (v) ${\mathrm{OH}}_{32}$ - ${\mathrm{OH}}_{40}$ in yellow. By inspecting the changes in these distances, one can gain insights into the structural rearrangements that occur in the system.

The distances between the ${\mathrm{OG}}_{35}$ and Mg in all substates of state 2, as shown by the violet line in Figure 2, are very similar, measuring approximately 2.0 Å. These values closely match the measurements obtained from the crystal structures of state 2, such as the substate $2_{OX}$ with the PDB identification 3K8Y [54]. This suggests that the changes in the ${\mathrm{OG}}_{35}$ -Mg distance within state 2 are consistently maintained across different substates.

**Figure 4:**
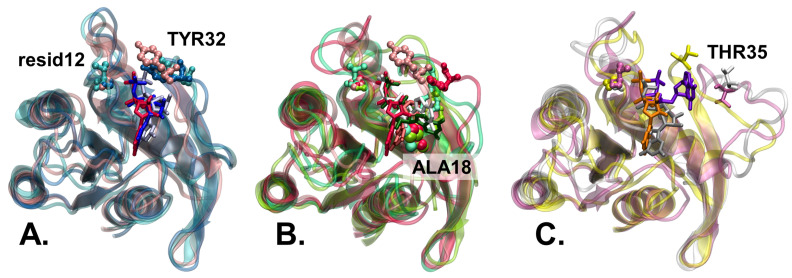
**Overlap of N-Ras structures in different (sub)states.** Protein G-domain secondary structure in transparent cartoon representation, Tyr32 and Gly/Val12 in stick-and-balls representation, Thr35 in licorice representation and Ala18 in balls representation are colored in the first color in paranthesis for each subfigure, while GTP in licorice representation is colored in the second listed color. (**A**). Substate $2_{OX}$ in membrane WT (dark blue/blue) and G12V (cyan/light blue) together with the 5UHV N-Ras crystal structure (pink/red) [52]. (**B**). Substate $2_{OY}$ in membrane WT (green/dark green) and G12V (light green/white) together with substate $2_{OZ}$ (red/light red). Ala18, shown only in this subfigure, highlights the different orientations of Tyr32 in the two substates. (**C**). State 1 in membrane WT (white/gray) and G12V (pink/mauve) together with the 4EFL H-Ras state 1 crystallographic structure (yellow/orange) [41]. Thr35 is shown instead of Tyr32 in this subfigure, to highlight its departure from the GTP-bound ${\mathrm{Mg}}^{2+}$ ions in state 1.

In contrast, state 1 is distinguished by the breaking of the hydrogen bond between the ${\mathrm{Mg}}^{2+}$ ion and Thr35. Consequently, there is a significant increase in the distance between ${\mathrm{OG}}_{35}$ and Mg, a change that has been observed in the crystal structure of state 1, as evidenced by the 4EFL crystal structure [41] (see Figure 4C). The identification of this alteration in the ${\mathrm{OG}}_{35}$ -Mg distance serves to further distinguish state 1 from state 2 and highlights the distinct molecular characteristics of each state.

The distance between the $O_{12}$ - $O_{\gamma }$ of GTP remains constant at around 5.3 Å in all the substates of state 2 of the WT systems. This value is relatively close to the distance observed in the crystal structure of the substate $2_{OX}$ of H-Ras (3K8Y [54]). However, when the G12V mutation is present, this distance is shifted, increasing to values above 8 Å. This shift is attributed to the significantly larger size of the Val12 residue compared to the wild-type Gly12 residue (see Figure 4A).

In substate $2_{OX}$ , the ${\mathrm{OH}}_{32}$ - $O_{\gamma }$ distance fluctuates around 2.7 Å. On the other hand, substate $2_{OZ}$ displays larger fluctuations in the ${\mathrm{OH}}_{32}$ - $O_{\gamma }$ distance, which centers around 7.5 Å. These fluctuations are due to the repositioning of the Tyr32 residue toward the bulk of the protein. This repositioning could be connected to the hydrolysis process associated with substate $2_{OZ}$ [43]. The newly identified substate $2_{OY}$ ’s orientation leads to the farthest positioning of the Tyr32’s terminal ring oxygen atom (OH) from the γ phosphate group of GTP, resulting in an ${\mathrm{OH}}_{32}$ - $O_{\gamma }$ distance of approximately 11.3 Å. In state 1, the switch I loop moves away from the GTP, and the breaking of the Thr35-Mg hydrogen bond causes a significant increase in the ${\mathrm{OH}}_{32}$ - $O_{\gamma }$ distance as compared to other substates. Overall, these findings highlight the dynamic nature of the ${\mathrm{OH}}_{32}$ - $O_{\gamma }$ distance and its crucial role in characterizing all the (sub)states of Ras proteins.

The conformation of substate $2_{OY}$ is unique due to the specific positioning of the Tyr32 residue toward Ala18. This particular orientation is characterized by the remarkably short ${\mathrm{OH}}_{32}$ - $O_{18}$ distance, measuring only 4.3 Å. In sharp contrast, the other substates exhibit significantly larger distances, exceeding 12 Å (see Figure 4B).

To closely monitor the changes of switch I, we calculated the distance ${\mathrm{OH}}_{32}$ - ${\mathrm{OH}}_{40}$ . In state 1, our simulations show a wide range of values. However, the repositioning of Tyr40 close to Tyr32 (the ${\mathrm{OH}}_{32}$ - ${\mathrm{OH}}_{40}$ distance value becomes significantly smaller) leads to the destabilization of Thr35 and determines the bond between the magnesium ion and the side chain oxygen of Thr35 to rupture. This change promotes the protein’s conversion from substate $2_{OX}$ into state 1, as depicted in the 4EFL [41] crystal structure ( ${\mathrm{OH}}_{32}$ - ${\mathrm{OH}}_{40}$ distance around 4.4 Å). The simulations do not show any important changes in switch I for the OX and OZ substates (the distance value is around 11.6 Å).

From a different perspective, we also focused our attention on characterizing the membrane-bound protein orientation with respect to the stretch of the HVR domain using two reaction coordinates (RCs) equivalent to those employed on G12V K-Ras [55]: (1) the angle between a vector along the β 1 sheet (residues 2–5) and the membrane normal (angle), and (2) the distance between C_α_ atoms of E132 on the α 4 helix and L184 on the HVR domain (dist). From the contour plots based on these two RCs (see Figure S7), one can observe that G12V mutations are accompanied by significant deviations or changes from the inherent dynamics characterized by these two RCs.

For WT, the substates OY and OZ of state 2 exhibit a more specific positioning of the G domain orientation with respect to a fairly compact HVR. Substate $2_{OX}$ overlaps with the other two but also exhibits a medium-stretched (and specific) HVR and a conformational connection “pathway” toward a very narrow HVR and specific orientation of state 1, consistent with state 1 acting as a “pool” for the WT active state 2 [43,56]. However, with the G12V mutation, these RCs become less specific for state 1 and substate $2_{OY}$ , with wide ranges of G-domain orientations and HVR distances being sampled. Substate $2_{OZ}$ is not sampled, and in substate $2_{OX}$ , the conformations sampled become selective for a medium-stretched HVR and a G domain orientation parallel to the membrane. Hence, the conformational overlap sampled in our simulations between state 1 and substate $2_{OX}$ becomes practically nonexistent. These findings suggest that the G12V mutation has an impact on the orientation of the G domain with respect to the membrane, thereby altering the protein’s interaction with the membrane.

### 2.2. Dynamics and Distribution of (Sub)States

The gray bars in Figure 2A(d) show that state 1 is the most populated state (∼40%), while substate $2_{OX}$ and substate $2_{OY}$ correspond to ∼30% and ∼20%, respectively. This is consistent with the recent attribution of state 1 as a “pool” for the “active” state 2 in the wild-type Ras [43,56]. Although substate $2_{OZ}$ is present only ∼3% of the time, it is crucial in the hydrolysis process, as it corresponds to the GAP-binding conformation [43].

Apart from the sidechain orientation, the three substates of state 2 can also be quantitatively distinguished by their root mean square deviation (RMSD) values with respect to the Ras crystal structure identified to be in substate $2_{OX}$ (3K8Y [54]) (see Figure S1). The RMSD plot also shows the sharp transitions between the substates, when they occur directly (details are in the Supplementary Material).

The overlay of the average structures for state 1 and state 2 shows significant variation at the loop of the SI region (see Figure S2). However, the core structure of the N-Ras protein remains very stable in all simulations, both in solution and membrane-bound, with C_α_ root mean square deviation (RMSD) less than 1.4 Å from the corresponding crystal structure (Figure S3) and averages from 0.94 Åto 1.15 Å. Nevertheless, significant differences are noted at switch I (SI) and switch II (SII) regions (see Figure S4), with mean RMSD values of 3.78 Å–6.77 Å and 4.27 Å–5.64 Å, respectively.

**Figure 5:**
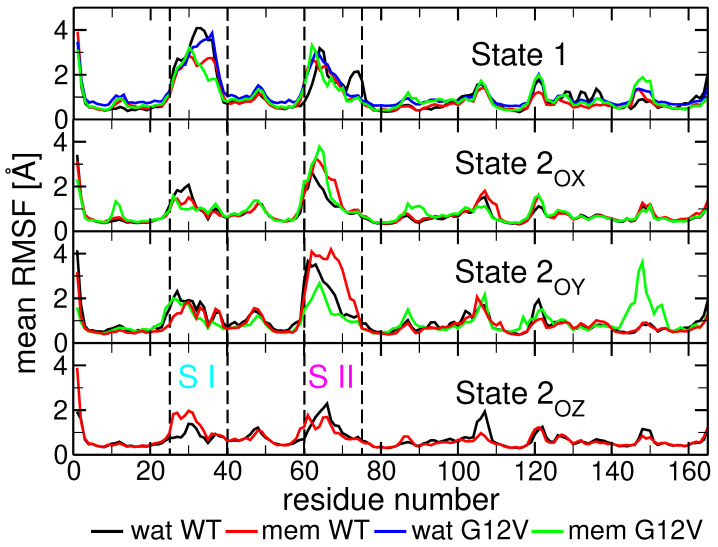
**Time-averaged RMSF of each residue’s C_α_ calculated for all (sub)states.** RMSFs were calculated for both WT (black line without membrane and red line membrane-bound) and G12V (blue line without membrane and green line membrane-bound) conformations. The locations of the switch I (SI in cyan) and switch II (SII in magenta) regions are also displayed between dashed boxes.

These deviations are reflected in the C_α_ root mean square fluctuation (RMSF) calculated for each residue, and time-weight averaged over several trajectories for each (sub)state (see Figure 5). State 1 is characterized by overall larger fluctuations of both switches than state 2 in general, and larger fluctuations of SI compared to SII, unlike state 2. The RMSF plots for WT in solution only (black line in Figure 5) show some small differences between the substates of state 2 (see Figure S5), consisting of (i) the lowest fluctuations for SI and SII regions of substate $2_{OZ}$ (which is consistent with the GAP-binding state corresponding structure), and higher on residues 105–107; and (ii) fluctuations for the SII region of substate $2_{OY}$ comparable to those of state 1 (i.e., 50–100%, larger than on the other two substates of state 2), but only on residues 60–69.

**Figure 6:**
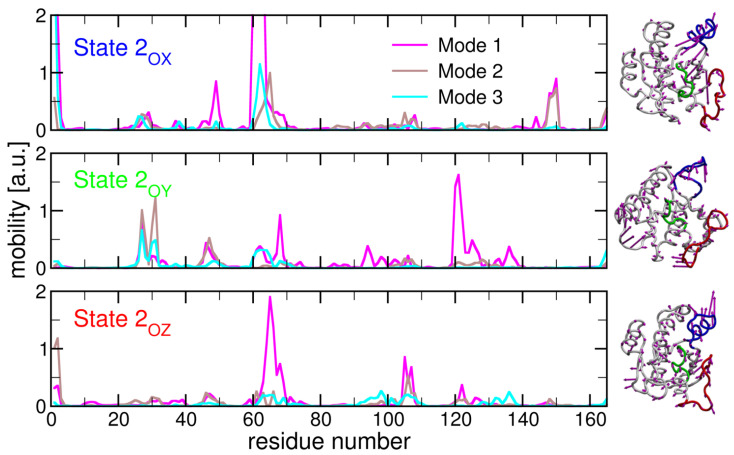
**Individual residue mobility in the principal component analysis for all (sub)states.** PCA from the MD trajectory was performed with the NMWiz plugin [57] in VMD [58] for WT in solution (i.e., without membrane). Of the first ten, three major modes are displayed (with magenta, brown, and cyan lines, respectively), separately for substate $2_{OX}$ (top), substate $2_{OY}$ (center), and substate $2_{OZ}$ (bottom), respectively. The normal modes of the first eigenvector are represented on the three protein insets for each substate.

The PCA and cross-correlation analysis allowed us to identify the key motions and interactions within N-Ras (see Figure 6 and Figure S6). The motions of switch I and switch II play a crucial role in the conformational changes of the N-Ras protein. The anti-correlated motion of these switch regions with other residues in the catalytic domain suggests that these regions may undergo opposite movements to facilitate the transition between different states. On the other hand, the reduced correlation observed in the active state suggests that other regions of the protein may become more rigid during activation (see Figure S6).

Figure S6 shows the cross-correlation matrices (obtained using the NMWiz plugin [57]) of residue pairs in the N-Ras protein for the three substates of state 2: substate $2_{OX}$ , substate $2_{OY}$ , and substate $2_{OZ}$ . We observed considerable correlations between residue pairs in substate $2_{OX}$ and substate $2_{OY}$ . The two switch regions, SI and SII, show stronger anti-correlated motion with other residues of the protein catalytic domain. In contrast, in the active substate of N-Ras (substate $2_{OZ}$ ), most cross-correlation values of residue pairs are close to 0, indicating a reduced correlation of these residue pairs.

To verify which motions could account for RMSD variations, we performed a (PCA) from the MD trajectory. Results for the first 10 eigenvectors confirm that the greatest motions were contained within the first 3 eigenvectors and they indeed came from Switch I and Switch II (see Figure 6). These regions have been described as highly flexible, as noticed by differences in the two crystallographic structures for different states [41,52]. However, this fact does not exclude the possible existence of small but significant motions that may be important for the transition between sub(states) (Figure 6).

### 2.3. Influence of Membrane on the N-Ras Conformational (Sub)States

The presence of the membrane leads to smaller fluctuations in state 1, especially in the SI region, due to the attachment of the proteins’ α -helices 2 and 4 to the membrane, along with the increased stability of the P-loop. While significant in the dynamics of state 1, the membrane’s presence does not seem to affect the ratio of time spent by N-Ras in state 1 during our simulations (see Figure 2A,B(d)).

Substate $2_{OY}$ is barely present (∼6%) when membrane-bound for N-Ras WT as compared to the ∼20% in solution only (i.e., without the membrane). In contrast, the presence of the membrane increases the stability of substate $2_{OX}$ as being the dominant substate for N-Ras WT (∼55% as opposed to ∼25% in solution only). Although present in a small and similar percentage (∼2%) as in the absence of the membrane, substate $2_{OZ}$ maintains its utmost importance due to its correspondence to the GAP-binding state [43].

These results suggest that the membrane plays a crucial role in modulating both the N-Ras dynamics of states and substates, and (mostly) the distributions of substate $2_{OX}$ and substate $2_{OY}$ sampled during our simulations (see Figure 2A vs. Figure 2B).

### 2.4. Influence of G12V Mutation on the N-Ras Conformational (Sub)States

The presence of state 1 increases to a majority of approximately 95% of the time (see Figure 2A(d)). This indicates a significant decrease in Thr35 binding stability to ${\mathrm{Mg}}^{2+}$ once the mutation occurs in the absence of the membrane (i.e., in solution only). When membrane-bound, on the other hand, both WT and the G12V mutation are sampled at about the same time in state 1.

Despite the oncogenic nature of the G12V mutation (and, therefore, its high stability), none of the substates of the "active" state 2 is present in the absence of the membrane (i.e., in solution only). This underscores the crucial importance of the protein–membrane interaction in the oncogenic mutation [22,59,60].

Due to the much larger size of Val compared to Gly in membrane-bound systems, the G12V mutation of N-Ras (see Figure 4) significantly reduces the presence of the protein in substate $2_{OX}$ from ∼55% in WT to only∼10% in G12V. Furthermore, the sampling time of substate $2_{OY}$ is increased five-fold, from ∼6% for WT to ∼30% for G12V (see Figure 2B(d)).

Perhaps the most important effect of the G12V mutation is on substate $2_{OZ}$ , which is not found in either membrane-free (i.e., solution-only) or membrane-bound systems. This is extremely important since the substate $2_{OZ}$ conformation corresponds to the GAP-bound conformation. The lack of sampling of this substate suggests the appearance of a significant energy barrier that most likely eliminates (or at least significantly reduces) enhanced GTP hydrolysis, rendering G12V into an oncogenic mutation.

### 2.5. Role of Ions

In substate $2_{OX}$ , the distance between ${\mathrm{OH}}_{32}$ – $O_{\gamma }$ of GTP is the smallest; hence, the accessibility of the ions to the GTP pocket is reduced (on average, only 0.42 ions). The reorientation of the plane of the hydrophobic ring of Tyr32 in substate $2_{OZ}$ breaks the ${\mathrm{OH}}_{32}$ – $O_{\gamma }$ hydrogen bond. The γ phosphate reforms its hydrogen bond with a water molecule. This way, the access of ions to the GTP pocket is blocked (see Table 1). The large change in the orientation of Tyr32 toward Ala18 naturally enhances the accessibility of ions (0.75). In state 1, the GTP pocket is open due to the shift of the SI loop, allowing a large number of ions to freely move in the pocket vicinity (∼1.7). When the G12V mutation occurs, the P-loop is shifted (by Val12), and therefore, the number of ions accessing the enlarged GTP pocket is significantly increased (+0.6–+0.8). Since in substate $2_{OY}$ Tyr32 is oriented along switch I toward Ala18, the water and ion accessibility is conserved (see Table S1).

This increase in the number of ions most definitely should impact the environment’s electrostatics and, therefore, the distribution of the conformational states. Our observations are in agreement with previous findings of an increased number of ${\mathrm{Na}}^{+}$ ions correlated to conformational changes due to mutations of Gln61 [46] or to the phosphorylation of Tyr32 [61].

## 3. Discussion

Overall, our findings suggest that the conformational dynamics of Ras proteins, specifically N-Ras, play a critical role in their oncogenicity and interaction with the cell membrane. The crystallographic structures of Ras proteins have revealed the existence of multiple conformational states, indicating the flexibility of these proteins. Among the GTP-bound states, two main states have been identified: the “inactive” state 1 and the “active” state 2. Recent studies on H-Ras have shown that state 2 can exist in two substates, distinguished by the orientation of Tyr32 toward the GTP-bound pocket or outwards [43]. In this study, we investigated the conformational dynamics of N-Ras and discovered an additional substate of state 2. This substate is characterized by the orientation of Tyr32 toward Ala18 and in parallel to the GTP-bound pocket.

Interestingly, we found that this substate is highly sampled in the G12V mutation of N-Ras in the membrane, which is commonly associated with oncogenicity, but barely present in the wild-type form. Moreover, our molecular dynamics simulations showed that the membrane-binding of N-Ras prevents the sampling of substate $2_{OY}$ and favors the sampling of substate $2_{OX}$ . Therefore, we believe that in WT, the membrane’s role is also to evade this specific substate $2_{OY}$ , and enhance N-Ras’ transition into the predominant substate $2_{OX}$ , previously identified as playing a key role in the conformational transition to the conformation substate $2_{OZ}$ (interacting with GAP’s arginine finger) [43].

The G12V mutation, on the other hand, has the opposite effect, minimizing substate $2_{OX}$ sampling and drastically increasing the sampling of substate $2_{OY}$ , rendering this mutation oncogenic. Interestingly, we observed that the G12V mutation in the absence of the membrane hinders the sampling of the substate related to the binding of the GTPase-activating protein (GAP) (substate $2_{OZ}$ ), and remains in state 1 until the end of our simulations.

In conclusion, our study reveals that the conformationally “active” state 2 of Ras exhibits three distinct substates, and highlights the significance of conformational dynamics and the influence of the cell membrane in Ras protein function and oncogenicity. Understanding the conformational landscape and membrane interactions of Ras proteins may provide valuable insights for the development of targeted therapies against Ras-driven cancers.

## 4. Methods

Using the accession number 5UHV [52] from the Protein Data Bank [62], the initial coordinates for the simulations were derived from crystal structures. Specifically, for the simulations involving the N-Ras-GTP complex, a refined crystal structure was selected as the reference. The N-Ras structure (residues 1–165) was altered using Chimera [63] to create a mutant structure for the N-Ras (G12V) G domain using the Dunbrack rotamer library [64], and the hypervariable region was created using the “Interactive peptide chains modeling” molefacture plugin from VMD [58]. The GTP molecule in the active site was left in the same location as the GTP analog in the original structure, and the γ -phosphate was manually substituted for the terminal group of the GTP analog found in the crystal structure to create a GTP ligand. Coordinates for crystal magnesium were overlaid on top of the magnesium ion center. The lipid bilayer structures were made using Membrane Builder [65]. Following the insertion of the protein into one leaflet, the system was neutralized and solvated with TIP3P water [66] to a physiological concentration of 0.15 mol/L of ${\mathrm{Na}}^{+}$ ${\mathrm{Cl}}^{-}$ .

N-Ras proteins form functional nanoclusters that are preferentially localized at the border of liquid-disordered ( $L_{d}$ ) and liquid-ordered ( $L_{o}$ ) domains [30]. The minimal membrane model required to obtain such phase separation is a ternary lipid bilayer with an unsaturated tailed phospholipid, a saturated tail phospholipid, and cholesterol [30]. For our mammalian membrane model, we used a ternary lipid bilayer formed of 160 DPPC (di-16:0-PC), 96 DOPC (di-18:1-PC), and 64 CHOL (cholesterol) (i.e., in 5:3:2 ratio), as in our previous studies [36,37].

Two separate types of atomistic molecular dynamics simulations were conducted using the NAMD 2.9 simulation program [67] to study the GTP-bound full-length N-Ras wild-type (WT) and mutant (G12V) proteins (details are in Table S2). The CHARMM36(m) force field for both lipids [68] and proteins [69,70] was employed in all our simulations. In our model systems, the guanosine triphosphate (GTP)-bound N-Ras protein model (see Figure 1A,B) was either solvated in water at a physiological ionic concentration (without the anchor and without the membrane) or inserted with its (natural) post-translationally lipid-modified anchors (palmitoylated Cys181–PAL and farnesylated Cys186–FAR) into our fully solvated membrane model (see Figure 1C) (membrane-bound) [55,71]. The two preferred orientations of Ras proteins with respect to the membrane were used as starting configurations in our N-Ras simulations [22].

The simulations were carried out using a standardized protocol. Firstly, each system was minimized for 5000 steps using a conjugate gradient energy minimization-based algorithm [72]. Then, a 100 ps warm-up was conducted, increasing the temperature from 0 to 300 K at a rate of 3 K/ps, followed by a 50 ps 300 K temperature coupling. Next, a 350 ps constrained equilibration was performed, only along the normal to the membrane direction, at 300 K. Finally, a production run of hundreds of ns was executed for each simulation using a step cycle of 20 and a 2 fs integration timestep, in conjunction with constraints applied to all bonds involving hydrogen using the SHAKE algorithm [73,74]. For unbound proteins (i.e., in a water solution only), the same protocol was used without inserting the protein into the membrane.

For the calculation of long-range electrostatic interactions, the particle mesh Ewald method [75] was employed. A spacing of 1 Å and a cutoff distance of 12 Å were used to accurately account for the long-range interactions between charged particles. To maintain a constant temperature of 300 K, a Langevin thermostat [76] was utilized during all simulations, with a damping coefficient of 1 ${\mathrm{ps}}^{-1}$ . Additionally, the pressure was controlled using a Langevin piston [77] at 1 atm, allowing for the simulation of the system under constant pressure conditions. These measures help create a stable and realistic environment for studying the behavior and properties of the simulated system.

## Figures and Tables

**Table 1:** The table contains the average number of ${\mathrm{Na}}^{+}$ ions (and their corresponding standard deviations) found in the vicinity (within 4.5 Å) of N-Ras’ GTP molecule for each of the four (sub)states (state 1 and state 2, with its three substates—OX, OY, OZ).

Location	Type	Avg. Number of ${\mathrm{Na}}^{+}$ Ions and Std. Dev.			
		**State 1**	**State 2_*OX*_**	**State 2_*OY*_**	**State 2_*OZ*_**
**A.**	**WT**	2.53 ± 1.52	0.56 ± 0.58	0.40 ± 0.61	0.15 ± 0.36
**sol. only**	**G12V**	1.56 ± 0.85	-	-	-
**B.**	**WT**	1.72 ± 0.58	0.42 ± 0.52	0.75 ± 0.58	0.06 ± 0.24
**membr.-bound**	**G12V**	2.30 ± 0.82	1.23 ± 0.65	0.88 ± 0.55	-

## Data Availability

All data published in this paper are within the manuscript or the Supplementary Material.

## Supplementary Materials

The following supporting information can be downloaded at: www.mdpi.com/xxx/s1.

## Abbreviations

The following abbreviations are used in this manuscript:

GTP	guanosine triphosphate
GDP	guanosine diphosphate
Ras	rat sarcoma virus
G-domain	Ras guanine nucleotide-binding domain
