# Supplementary material for: GTP-Bound N-Ras Conformational States and Substates Are Modulated by Membrane and Point Mutation
Source: Int J Mol Sci. 2024 Jan 24;25(3):0. doi: 10.3390/ijms25031430 (PMC11154311; doi:10.3390/ijms25031430)
Supplement: Supplementary file 1 [file ijms-25-01430-s001.zip › ijms-2772934-supplementary.pdf]

# Supplementary Materials: GTP-bound N-Ras Conformational States and Substates are Modulated by Membrane and Point Mutation

**Alexandra Farcas [1] and Lorant Janosi [1,*]**

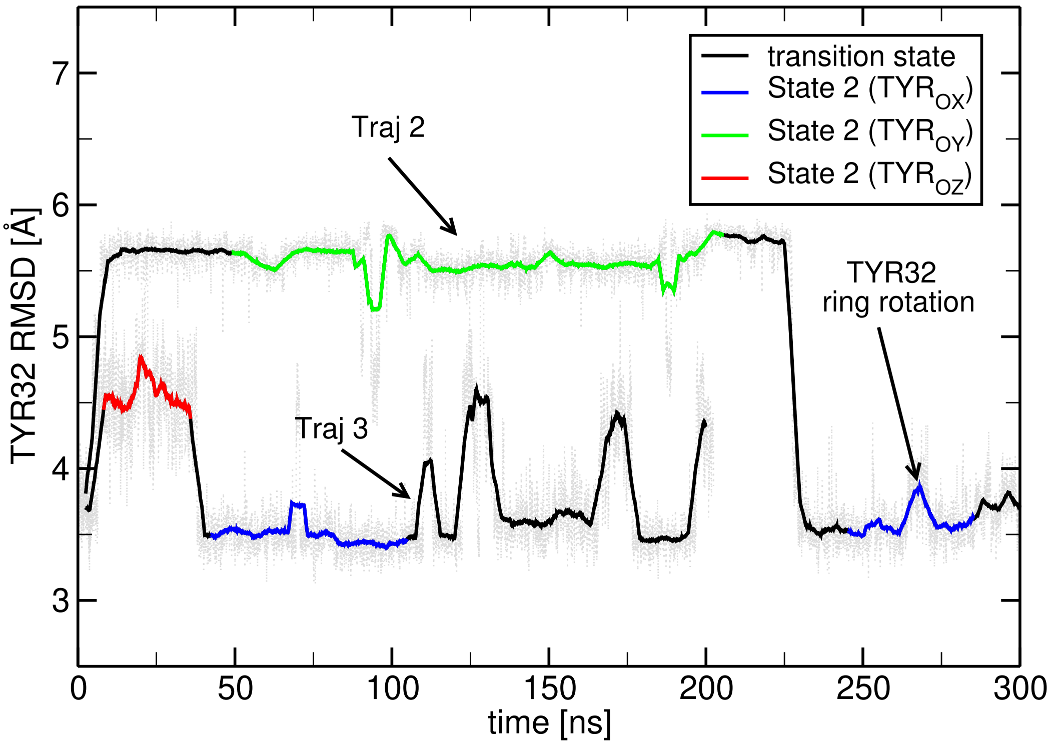


**Figure S1. Root mean square deviation (RMSD) of Tyr32 N-Ras residue for the three substates of state 2.** As an alternative quantitative characterization of the three substates of state 2, the RMSD was calculated for Tyr32 with respect to the substate $2_{OX}$ crystal structure (PDB id 3K8Y) for two simulations of N-Ras WT in solution only (i.e., for trajectories 'traj 2' and 'traj 3' from 'sol.only WT' in Tab. S2). Dotted gray lines represent the actual RMSD values, while the thick continuous lines are running averages, shown for clarity. Coloring is the following: black lines are for intermediate/transition states, blue line for substate $2_{OX}$ (averaged at $\sim$ 3.5 Å), green line for substate $2_{OY}$ (averaged at $\sim$ 5.6 Å), and red line for substate $2_{OZ}$ (averaged at $\sim$ 4.6 Å).
Notes: (i) The peak around 265 ns is also shown in blue (and not as a transition state) because it represents only a rotation of the Tyr32 ring, and not a change in its orientation, i.e., change in substate; (ii) RMSDs of Tyr32 were not displayed for 'traj 2' beyond $\sim$300 ns because N-Ras transits to state 1 and, therefore, the RMSD comparison to substates of state 2 is meaningless.

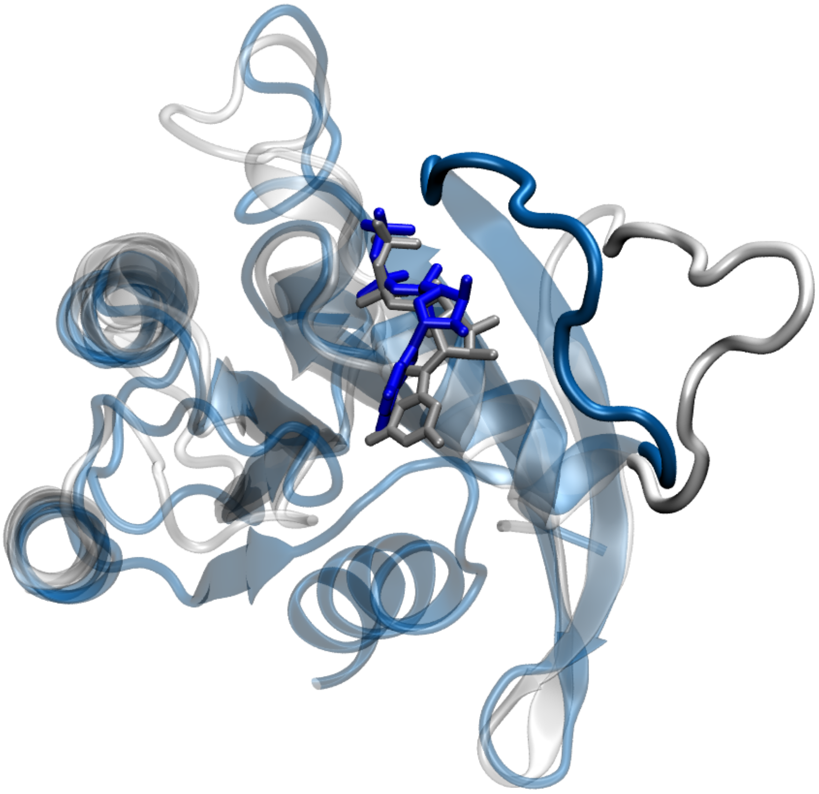

**Figure S2. Overlap of state 1 (white) and state 2 (blue) conformations of N-Ras protein.** The main difference between the two states is highlighted in the flexible region of SI (opaque cartoon representation). GTP orientations for the two states are presented in licorice representation.

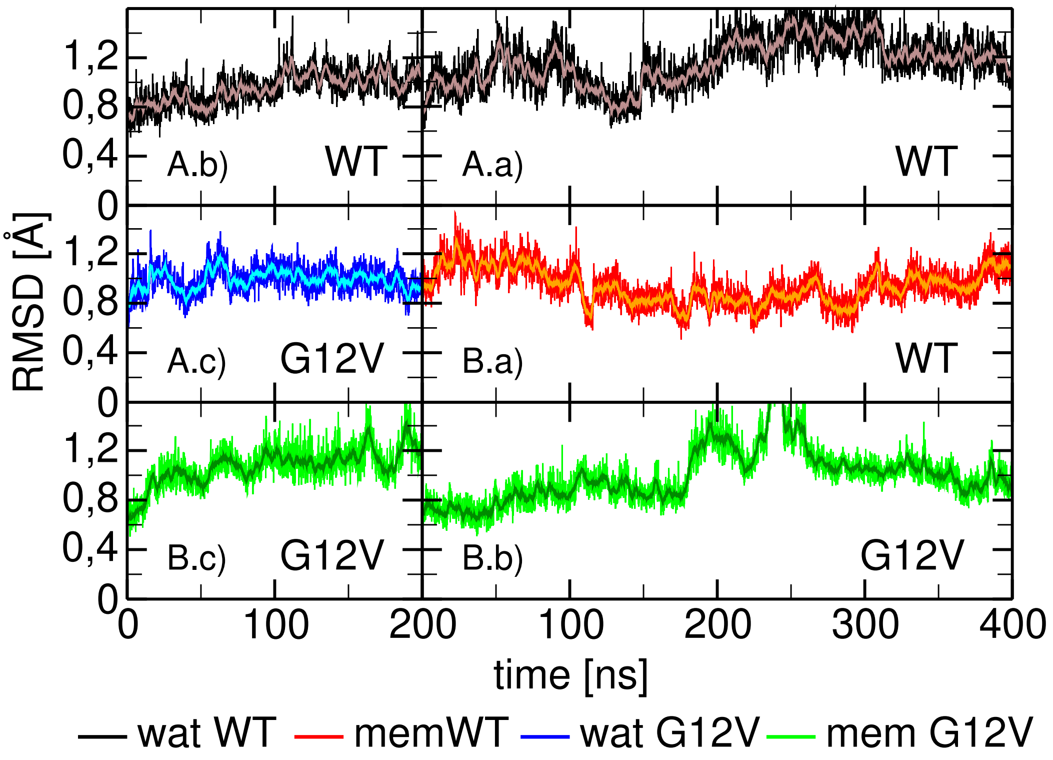


**Figure S3. Total root mean square deviation (RMSD) of N-Ras calculated over different simulations.** In order to check the stability of the N-Ras protein simulations, the RMSD was calculated with respect to the crystal structure of N-Ras (PDB id 5UHV), excluding the highly flexible regions. As examples, the 6 simulations presented in Fig. 2 are shown. Since not all simulations reached the 400 ns length, the position of each trajectory from Fig. 2 was rearranged, as labeled on the left-hand side (A—in solution only and B—membrane-bound). (In black are simulations in solution only of WT, in red are simulations with membrane of WT, in blue are simulations in solution only for G12V, and in green are simulations with the membrane of G12V). For clarity, the mean values are shown in contrasting lines. The average RMSD values ranged from 0.95 Å to 1.15 Å in solution only and from 0.94 Å to 1.07 Å in membrane-bound, and from 0.94 Å to 1.15 Å for WT and from 0.99 Å to 1.07 Å for G12V Å.

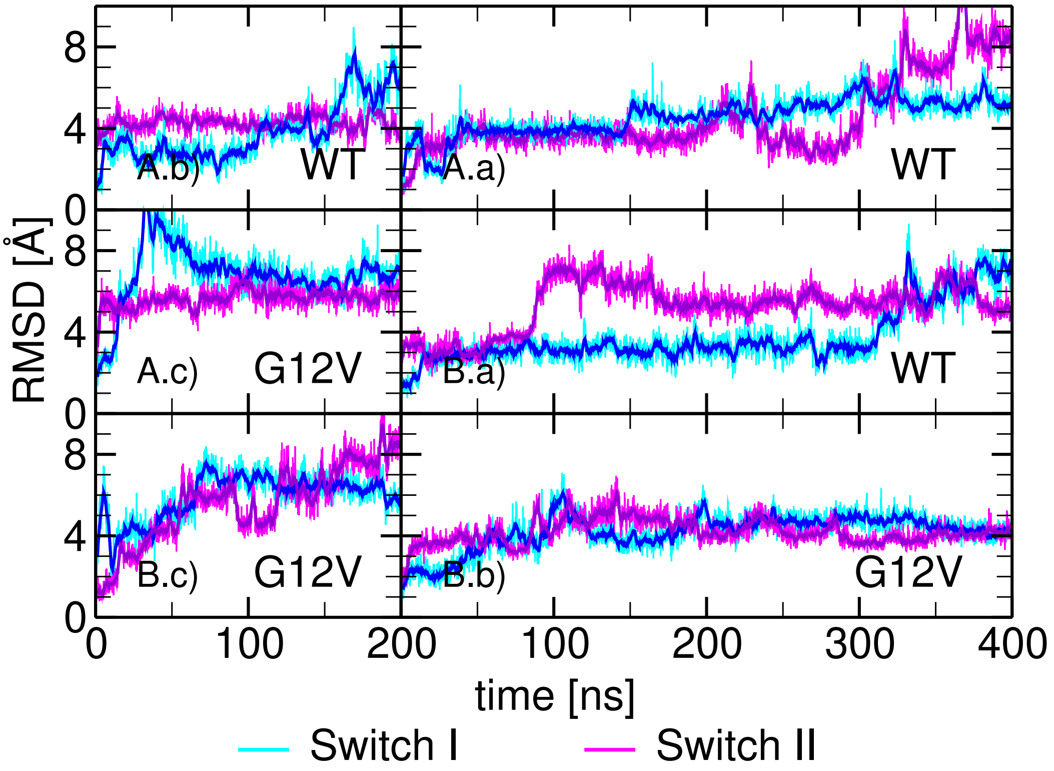


**Figure S4. Root mean square deviation (RMSD) of switch I (SI) and switch II (SII) regions of N-Ras.** Time evolution of the RMSD values of SI (calculated with respect to the crystal structure 5UHV) for the highly flexible regions, i.e., switch I (SI) (cyan) and switch II (SII) (violet) regions, respectively. The calculations were performed on the same set of simulations as those presented in Fig. 2 and Fig. S2. For clarity, the mean values are shown in contrasting lines.

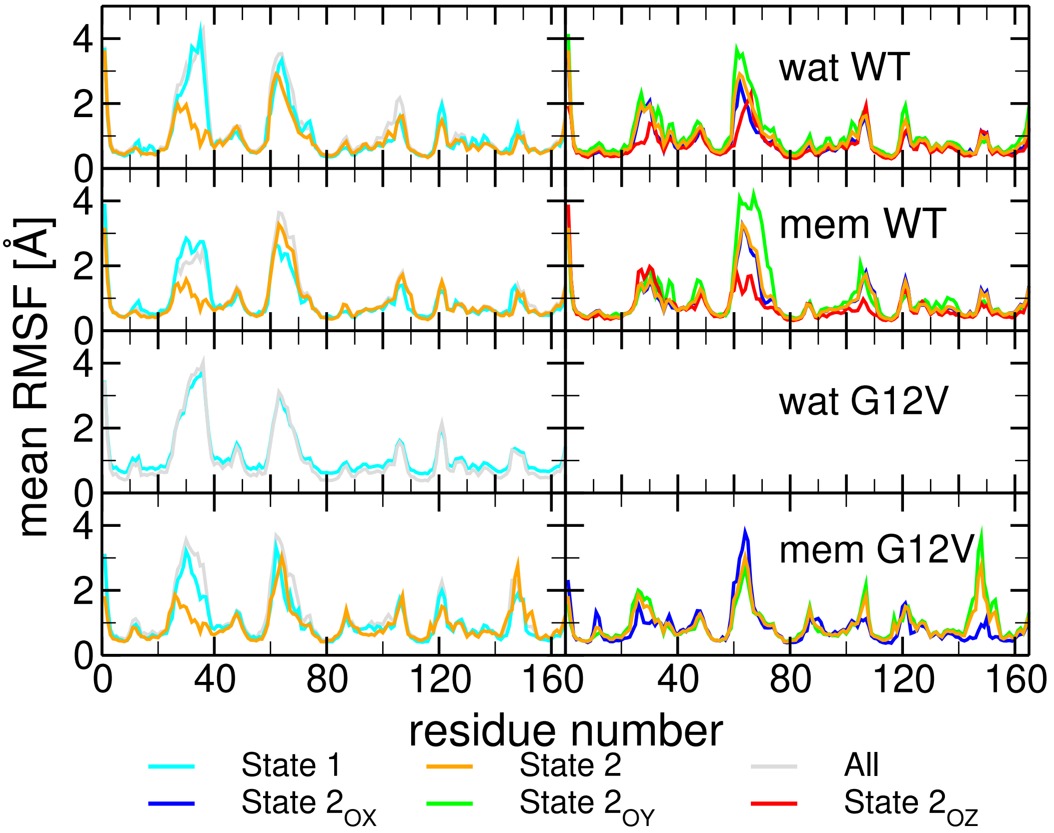


**Figure S5. Time-weighted average root mean square fluctuations (RMSFs) of N-Ras residues computed for each (sub)state.** The time-weighted average RMSF was computed for each type of simulation (in lines: in solution only WT, membrane-bound WT, solution only G12V, and membrane-bound G12V, respectively). The left-hand side column represents the mean RMSF computed over all trajectories (gray), state 1 (cyan), and state 2 (orange). On the right-hand side, state 2 (orange) is decomposed over each substate: substate $2_{OX}$ (blue), substate $2_{OY}$ (green), and substate $2_{OZ}$ (red), respectively.

Notes: (i) N-Ras did not exhibit state 2 (any of its substates) in solution-only G12V simulations; (ii) Please take note of the significantly different behavior between state 1 and state 2, between the substates of state 2, and how this behavior changes when N-Ras is anchored to the membrane (vs. free in solution), or when it is G12V-point-mutated (vs. wild type). More details are in the main text.

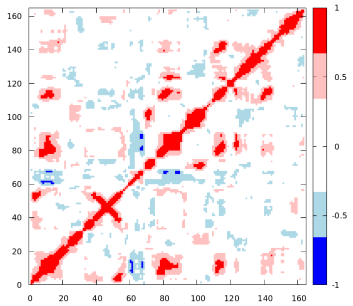

**(a)** SubSt $2_{OX}$ – WT membr.-bound Traj 1

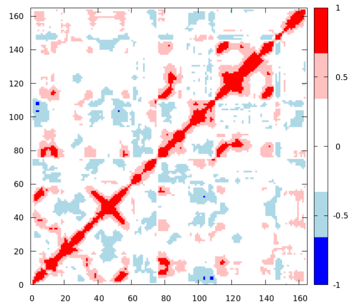

**(b)** SubSt $2_{OX}$ – WT membr.-bound Traj 2

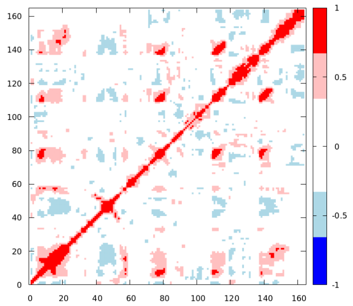

**(c)** SubSt $2_{OY}$ – WT in sol. Traj 2

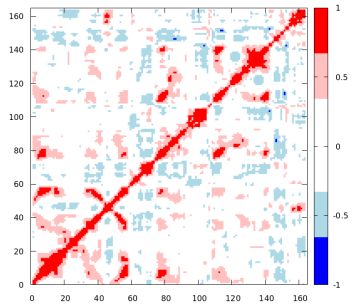

**(d)** SubSt $2_{OY}$ – G12V membr.-bound Traj 2

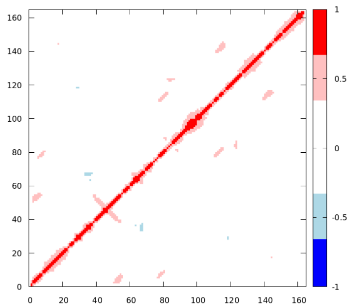

**(e)** SubSt $2_{OZ}$ – WT membr.-bound Traj 1

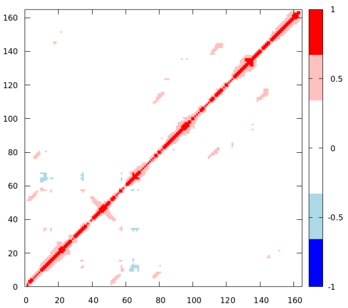

**(f)** SubSt $2_{OZ}$ – WT in sol. Traj 3

**Figure S6. Correlated motions of residue pairs in N-Ras.** The cross-correlation strength is colored, ranging from red (for Cij $\geq 0$, lockstep motions) to blue (for Cij $\leq 0$, anti-correlated motions).

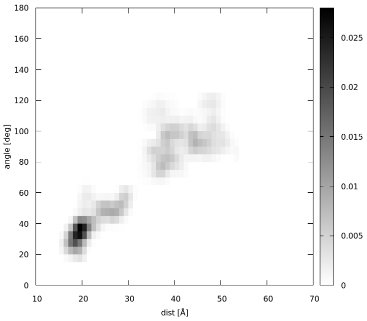


**(a)** St 1 WT

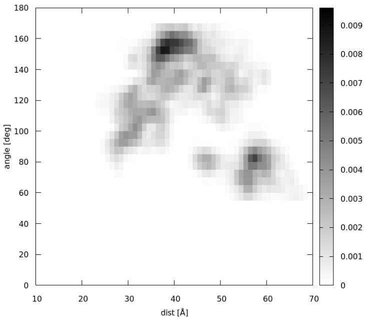


**(b)** St 1 G12V

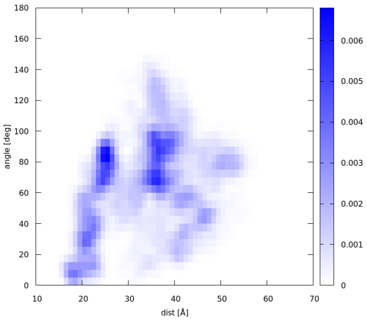


**(c)** SubSt $2_{OX}$ WT

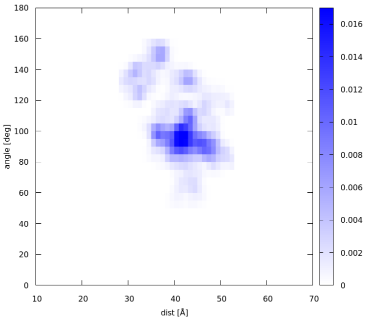


**(d)** SubSt $2_{OX}$ G12V

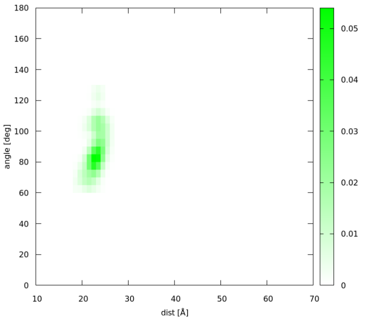


**(e)** SubSt $2_{OY}$ WT

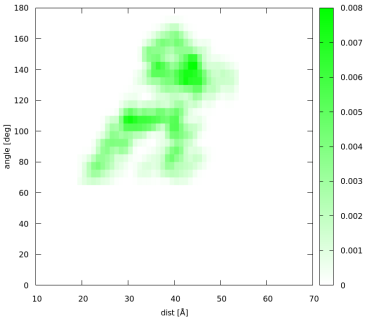


**(f)** SubSt $2_{OY}$ G12V

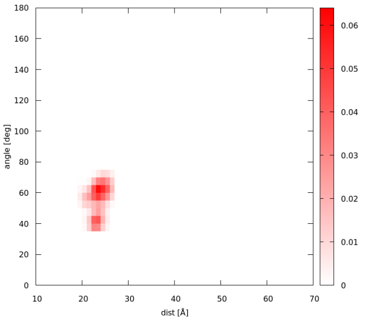


**(g)** SubSt $2_{OZ}$ WT

**(h)** SubSt $2_{OZ}$ G12V

**Figure S7. Contour plot depicting the angle relative to the membrane normal against the lipid anchor stretch distance.** The angle between a vector along $\beta 1$ (residues 2–5) and the membrane normal (angle) versus the distance between $C_\alpha$ atoms of E132 on $\alpha 4$ and L184 on the lipid anchor (dist).

**Table S1.** Table containing the average number of water molecules (and their corresponding standard deviations) found in the vicinity (water oxygen within 3.1 Å) of the N-Ras' GTP molecule for each of the four (sub)states (state 1 and state 2, with its three substates—OX, OY, and OZ).

| **Location** | **Type** | **Avg. number of water molecules and std. dev.** | | | |
|---|---|---|---|---|---|
| | | **State 1** | **State 2$_{OX}$** | **State 2$_{OY}$** | **State 2$_{OZ}$** |
| **A.** | **WT** | 9.78±2.64 | 5.50±1.21 | 7.10±1.31 | 7.88±0.95 |
| **sol. only** | **G12V** | 10.71±1.97 | - | - | - |
| **B.** | **WT** | 9.72±2.04 | 5.29±1.48 | 6.33±1.14 | 7.24±0.97 |
| **membr.-bound** | **G12V** | 11.03±1.58 | 8.51±1.20 | 6.86±1.36 | - |

**Table S2.** Table showing the total number of times (expressed in ns) that N-Ras exhibited each of the four (sub)states (state 1 and state 2, with its three substates—OX, OY, and OZ) for every type of simulation (described in the first column). These times are compared to the total simulation times for all the trajectories (last column). The simulation box sizes for the systems are (in Å): (i) sol. only: WT – 58 x 57 x 70, G12V – 61 x 56 x 66; (ii) membr.-bound: WT – 82 x 98 x 113, G12V – 83 x 96 x 119.

| Location | Type | Trajectory | Time [ns] | | | | |
|---|---|---|---|---|---|---|---|
| | | | **State 1** | **SubSt $2_{OX}$** | **SubSt $2_{OY}$** | **SubSt $2_{OZ}$** | **Simulated** |
| **A. sol.only** | **WT** | Traj 1 | 95 | 95 | 0 | 0 | 200 |
| | | Traj 2 | 117 | 40 | 157 | 0 | 400 |
| | | Traj 3 | 0 | 62 | 0 | 27 | 200 |
| | | **Total** | **212** | **197** | **157** | **27** | **800** |
| | **G12V** | Traj 1 | 200 | 0 | 0 | 0 | 200 |
| | | Traj 2 | 185 | 0 | 0 | 0 | 200 |
| | | Traj 3 | 189 | 0 | 0 | 0 | 200 |
| | | **Total** | **574** | **0** | **0** | **0** | **600** |
| **B. membr.-bound** | **WT** | Traj 1 | 74 | 270 | 0 | 39 | 400 |
| | | Traj 2 | 0 | 300 | 0 | 0 | 300 |
| | | Traj 3 | 0 | 200 | 0 | 0 | 200 |
| | | Traj 4 | 62 | 254 | 0 | 0 | 350 |
| | | Traj 5 | 0 | 142 | 136 | 0 | 300 |
| | | **Total** | **136** | **1166** | **136** | **39** | **1550** |
| | **G12V** | Traj 1 | 107 | 25 | 0 | 0 | 200 |
| | | Traj 2 | 0 | 0 | 30 | 0 | 350 |
| | | Traj 3 | 0 | 0 | 235 | 0 | 200 |
| | | Traj 4 | 90 | 143 | 0 | 0 | 300 |
| | | Traj 5 | 193 | 0 | 91 | 0 | 350 |
| | | **Total** | **390** | **168** | **356** | **0** | **1200** |
| | | **Overall** | | | | | **4150** |
